# Acid–base reaction-based dispersive solid phase extraction of favipiravir using biotin from biological samples prior to capillary electrophoresis analysis†

**DOI:** 10.1039/d3ra07356d

**Published:** 2024-06-19

**Authors:** Elnaz Safari, Behrouz Seyfinejad, Mir Ali Farajzadeh, Mohammad Reza Afshar Mogaddam, Mahboob Nemati

**Affiliations:** a Pharmaceutical and Food Control Department, Faculty of Pharmacy, Tabriz University of Medical Sciences Tabriz Iran nematim@tbzmed.ac.ir mahnema@gmail.com; b Pharmaceutical Analysis Research Center, Tabriz University of Medical Sciences Tabriz Iran; c Food and Drug Safety Research Center, Tabriz University of Medical Sciences Tabriz Iran Afsharmogaddam@tbzmed.ac.ir mr.afsharmogaddam@yahoo.com +98 4133344798 +98 4133372250; d Department of Analytical Chemistry, Faculty of Chemistry, University of Tabriz Tabriz Iran; e Engineering Faculty, Near East University 99138 Nicosia Mersin 10 North Cyprus Turkey

## Abstract

In this study, an acid–base reaction-based dispersive solid-phase extraction method was developed for the extraction of favipiravir from deionized water, plasma and urine samples prior to its determination using a capillary electrophoresis-diode array detector. The target analyte was extracted from the samples using biotin as a green adsorbent. To reach this goal, the pH of the solution was first adjusted to 9.0 (using borate buffer), and the ionic strength of the solution was enhanced by adding sodium chloride (2.5%, w/v). Thereafter, an appropriate amount of biotin was dissolved in the solution and a homogenous phase was obtained. By adding hydrochloric acid to the solution, an acid–base reaction occurs *via* protonation of biotin, which decreases its solubility. During this procedure, the analyte was adsorbed onto the tiny particles of the produced adsorbent dispersed into the solution. The resulting mixture was sonicated to facilitate the adsorption of the analyte onto the adsorbent surface. After the collection of biotin particles through centrifugation, the analyte was eluted using acetonitrile and then used in the determination stage. Under the optimal extraction conditions, the calibration curve was linear from 250 to 3000 ng mL^−1^ with a coefficient of determination of 0.9968. Low limit of detection, and quantification, good repeatability on the same day and different days (relative standard deviation ≤ 8.2%), and acceptable extraction recovery were accessed. The applicability of the method was examined by performing it on spiked plasma and urine samples, and its performance was verified.

## Abstract 2

An acid–base reaction-based dispersive solid-phase extraction method was developed for the extraction of favipiravir from deionized water, plasma and urine samples prior to its determination using a capillary electrophoresis-diode array detector.

## Graphical abstract

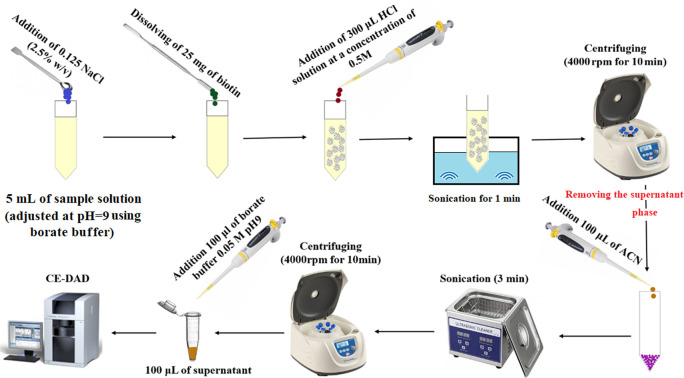

## 1. Introduction

Despite significant advances in the production of antiviral drugs, antibiotics, and vaccines, infectious diseases are still one of the leading causes of death worldwide, and epidemics of emerging infectious organisms pose a serious threat to human health.^1^ To combat emerging viruses and their mutated forms, the development of new therapies is needed. At present, coronavirus disease is a widespread concern around the world. Although different vaccines are currently available globally, discovering a new and specific antiviral drug against COVID-19 is a long and difficult task. One of these drugs is favipiravir, which has been first studied for the influenza virus and then for emerging pathogens such as Ebola and COVID-19 in humans.^2^ It was first used at the Wuhan Epidemic Center against SARS-CoV-2. With the spread of the epidemic in Europe, the drug was approved for emergency use in Italy and is used in most countries against COVID-19.

Favipiravir or 6-fluoro-3-hydroxypyrazine-2-carboxamide is an antiviral prodrug of the pyrazine class that selectively inhibits RNA polymerase in a wide variety of RNA-carrying viruses and inhibits virus replication.^3^ This prodrug has high bioavailability (approximately 94%) and a low volume of distribution.^4^ It requires active phosphorylation in tissues, and the main prodrug is metabolized to an inactive oxidative metabolite (T-705M1) *via* aldehyde oxidase.^2^ The pharmacokinetic complexities of this drug and the results of its different efficacies necessitate further studies to understand its correct dose in patients.^5^ Therefore, determination of the concentration profile of this drug in biological samples such as urine, plasma, and blood is necessary to prescribe the correct doses. Researchers have thus far used various methods such as high-performance liquid chromatography (HPLC)-tandem mass spectrometry,^6,7^ HPLC-ultraviolet detection,^8–10^ HPLC-mass spectrometry,^11^ and ultra-performance liquid chromatography-tandem mass spectrometry^12–14^ to determine the concentration of favipiravir in real samples. Capillary electrophoresis (CE) is another technique that can be used for drug analysis. CE is a robust separation and quantification technique that often offers higher resolution, shorter analysis times, and lower operating costs than conventional HPLC or gel electrophoresis.^15–23^ However, this technique as well as other separation-based techniques cannot be applied directly to the analysis of drugs in real samples due to the complexity of the real sample matrix and/or low concentration of the drug. Consequently, removing interferences and preconcentration of the analytes are essential before CE.^24^ The dispersive solid phase extraction (DSPE) method is known as an efficient extraction and clean-up method and is utilized in various matrices.^25^ This method is based on the dispersion of an adsorbent into the sample solution containing the analytes. The analytes are adsorbed physically or chemically on the adsorbent and extracted from the sample solution.^26^ By eluting the adsorbed analytes from the adsorbent surface with an eluent with volumes less than the sample solution, preconcentration occurs simultaneously. The contact surface area of the adsorbent with the sample solution plays a key role in DSPE and many attempts have been made to enhance it by using various stirring and shaking approaches. In recent years, *in situ* formation of the adsorbent in sample solution has attracted huge attention, because of the unlimited contact area between the adsorbent and sample solution.^27^

The chief aim of this study was to present a DSPE method for the extraction of favipiravir from biological samples prior to its analysis by CE. In the offered DSPE, the adsorbent was formed in the sample solution *via* an acid–base reaction by altering the solution pH. For this purpose, biotin was used as the adsorbent, which is soluble in alkaline pH. By adding a proper volume of an acid solution, biotin was re-precipitated in the sample solution as tiny particles. Performing this procedure led to the effective extraction of the analyte from the biological samples. The method does not require toxic solvents or compounds, and it can be considered an environmentally friendly method.

## 2. Materials and methods

### 2.1. Chemicals

Favipiravir was kindly gifted by Actoverco company (Karaj, Alborz Province). Biotin and folic acid, utilized as sorbents, were purchased from Sigma-Aldrich (St. Louis, Missouri, USA). The used inorganic compounds, sodium chloride, sodium hydroxide, and sodium tetraborate decahydrate as well as organic solvents acetone, acetonitrile (ACN), ethanol, and methanol were bought from Merck (Darmstadt, Germany). Hydrochloric acid solution (37%, w/w) was obtained from Dr Mojallali Co (Tehran, Iran). Deionized water, produced by Shahid Ghazi Pharmaceutical Company (Tabriz, Iran), was used for the preparation of the working solutions. A stock solution of favipiravir was prepared in methanol at a concentration of 500 mg L^−1^, and it was used in all optimization and validation steps for obtaining working solutions by diluting with deionized water.

### 2.2. CE instrumentation

CE determinations were carried out utilizing an HP 3D CE gadget (Hewlett-Packard, Palo Alto, CA, USA) equipped with a diode array detector (DAD). Instrumental control and information processing were performed utilizing ChemStation software (Agilent Innovations, Waldbronn, Germany). The capillary was from Agilent Co (Waldbronn, Germany; 75 μm i. d. × 40 cm (31.5 cm effective length). The capillary column was pretreated with NaOH (1 mol L^−1^), deionized water, and 50 mM borate buffer (as the background electrolyte (BGE)) for 30 min. The ideal conditions for electrophoretic separation were: BGE was 50 mM borate buffer at pH 9.0. The samples were injected at a hydrodynamic pressure of 50 mbar for 10 s. The separation voltage was +25 kV, the current was about 399 μA and the capillary was thermostated at 25 °C. The detection wavelength was 321 nm. BGE was sonicated and filtered through a PTFE syringe filter with a pore size of 0.20 μm. The pre-conditioning time was 2 min for sodium hydroxide, 2 min for deionized water, and 4 min for 50 mmol L^−1^ borate buffer with a pH of 9.0.

### 2.3. Real samples

In this method, two urine samples were obtained from healthy volunteers who did not consume the drug. The urine samples were diluted with deionized water before performing the extraction method. Two plasma samples were obtained from the Iranian Blood Transfusion Organization (Tabriz, Iran). They were used in the validation steps. The samples were kept in a freezer at −18 °C before analysis. One milliliter of plasma was mixed with 50 mg trichloroacetic acid, and after vortexing for 3 min, the mixture was centrifuged at 12 000 rpm for 5 min. The clear supernatant was diluted with deionized water, up to 5 mL.

### 2.4. DSPE procedure

**Fig. 1:**
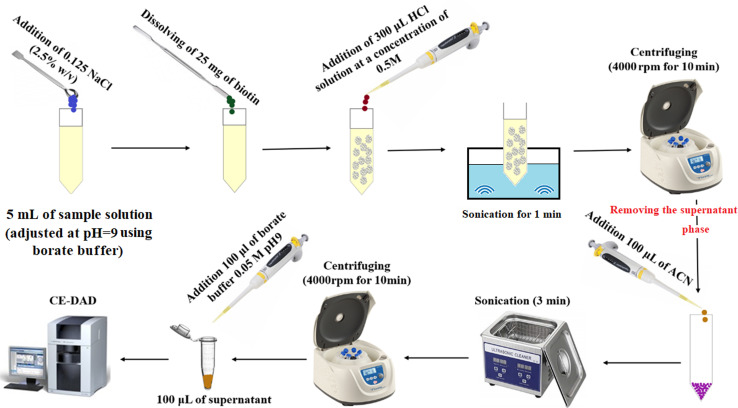
Extraction procedure steps.

Five milliliters of deionized water spiked with favipiravir at a concentration of 250 ng mL^−1^ or pretreated urine/plasma samples were poured into a glass test tube, and then its pH was adjusted to 9.0 using the borate buffer (50 m mol L^−1^). Then, 0.125 g (2.5%, w/v) sodium chloride was dissolved in the solution to adjust ionic strength. Then, 25 mg of biotin was added to the solution as the adsorbent and manually shaken to obtain a clear and homogenous solution. After that, 300 μL HCl solution (0.5 mol L^−1^) was added to the solution for protonation of biotin and decreasing its solubility. After sonication (1.0 min), the precipitated particles of biotin were collected at the bottom of the tube by centrifugation for 10 min at 5000 rpm. The supernatant was discarded, and the analyte was eluted with 100 μL ACN under sonication (for 3 min). The eluate was removed after centrifugation and mixed with 100 μL borate buffer (50 m mol L^−1^), and after filtration, it was analyzed using the CE-DAD system. The method steps are illustrated in Fig. 1.

## 3. Results and discussion

### 3.1. Selection of sorbent type and amount

**Fig. 2:**
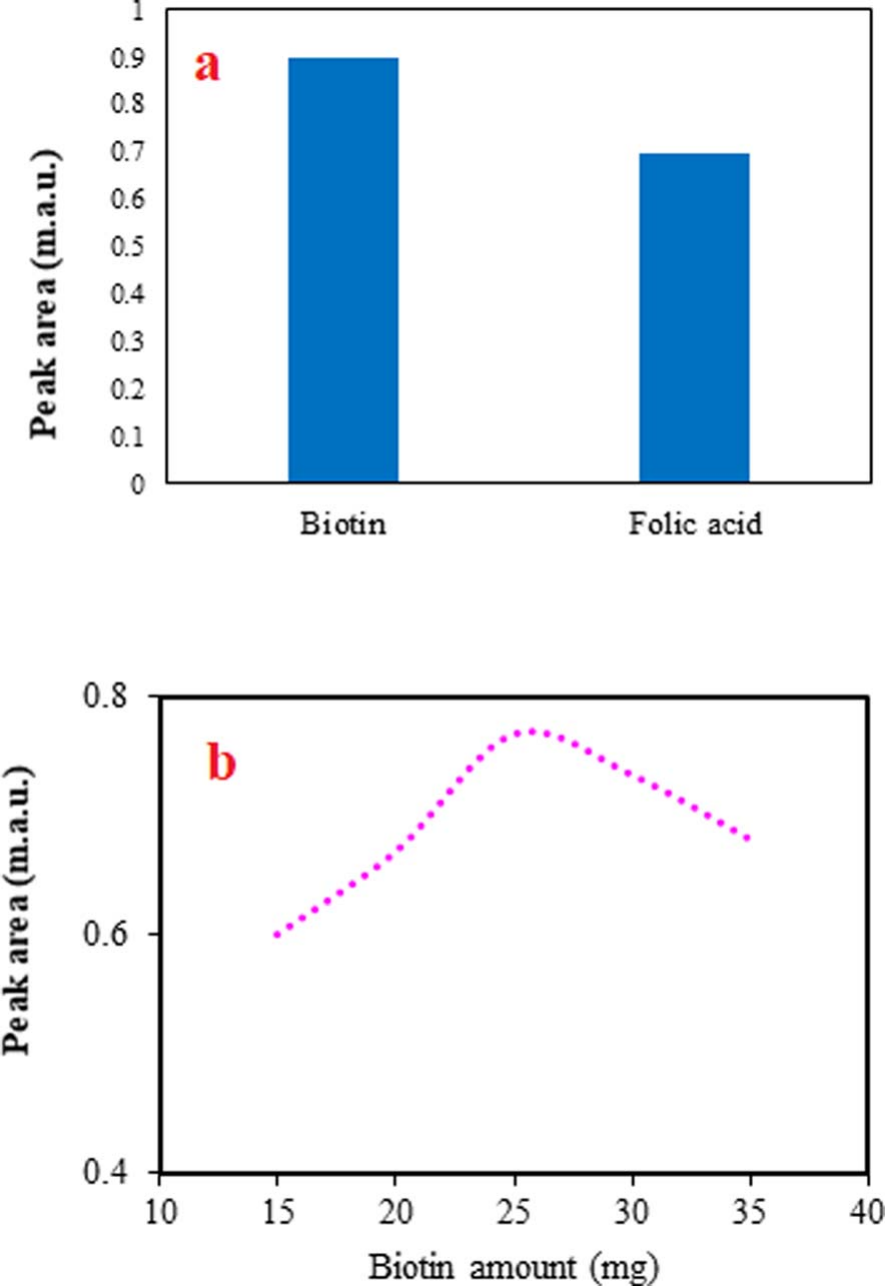
The effect of adsorbent type (a) and amount (b) on extraction efficiency. (a) Extraction conditions: sample, 5 mL deionized water spiked with favipiravir at 250 ng mL^−1^; adsorbent amount, 30 mg; agitation type (time), vortex (1 min); acid solution concentration (volume), 0.5 mol L^−1^ (300 μL); elution solvent (volume), ACN (200 μL); desorption time, 2 min; and centrifugation rate (time), 4000 rpm, 5 min. The error bars indicate the standard deviations of three repeated analyses. (b) Extraction conditions are the same as those discussed in the caption to Fig. 2a, except biotin was selected as the adsorbent.

The selection of a proper adsorbent to use in the developed DSPE method is the most significant step. The adsorbent should be soluble in an alkaline solution and re-precipitated with decreasing pH to acidic values. Considering these criteria and between the possible compounds, folic acid and biotin were chosen and applied in the method to extract favipiravir. The peak areas for the analyte using biotin and folic acid (Fig. 2a) depict that biotin was more effective than folic acid. It can be related to the better dispersion of biotin in the sample solution by changing pH and providing a high contact area. Therefore, biotin was chosen for the next steps.

In the DSPE method, various amounts of adsorbent provide different adsorption sites for the analyte and alter the extraction efficiency of the method. Usually, increasing the adsorbent amount improves the method's efficiency by increasing the extraction of more analytes. However, in several cases, increasing the adsorbent amount can have an adverse effect on the method because of the difficulties in the dispersion of the adsorbent particles into the solution and obtaining a limited contact area. In this study, the effect of the adsorbent amount was studied by altering its amount from 5 to 35 mg of biotin. The method was not applicable when the adsorbent was between 5 and 10 mg, since re-precipitation of it did not occur. The obtained data (Fig. 2b) show that increasing the biotin amount from 15 to 25 mg increases the efficiency of the proposed method, but adverse effect is observed after that. As a result, 25 mg of biotin was selected to continue the work.

### 3.2. Optimization of HCl solution concentration and volume

The present method is based on altering the solution pH from alkaline to acidic values because of the limited solubility of the adsorbent in acidic media and due to this HCl solution was used to reduce the solubility of biotin and change the solution pH. It is obvious that the concentration and volume of the used HCl solution have direct effects on the amount of biotin re-precipitated from the solution. Thus, HCl solution concentration was examined in the range of 0.3–1.0 mol L^−1^. It is notable that at concentrations less than 0.3 mol L^−1^, the method does not work. The experimental results (Fig. S1a†) illustrate that the peak area increases up to 0.5 mol L^−1^ and reaches constant values at higher concentrations. Increasing the analytical data is related to increasing the produced biotin particles and enhancing the adsorption sites. It is remarkable that at concentrations higher than 0.5 mol L^−1^, the separated amounts of biotin were constant, and due to this, the signals reached constant values. Thus, the following experiments were performed with HCl solution at a concentration of 0.5 mol L^−1^.

After obtaining the suitable concentration of HCl solution, its volume was optimized by performing various experiments at different volumes of HCl solutions in the range of 300–500 μL. The experimental results (Fig. S1b†) show that increasing the volume of the HCl solution has a negative effect on the method's efficiency. It may be related to increasing the sample solution volume and increasing the analyte solubility in the sample solution and decreasing the ratio of the sample solution to the extractant phase. To have high extraction efficiency, 300 μL was chosen as the optimum volume of HCl solution.

### 3.3. Selection of agitation type and time

To accelerate the extraction process, agitation of the sample solution after adding the HCl solution was carried out. In this work, the effect of agitation type was first evaluated by the use of vortexing or sonication, and the data (Fig. S2a†) confirmed the better role of sonication compared to vortexing. It can be related to more dispersion of the sorbent in the sample solution using sonication. Therefore, sonication was chosen in the other tests.

Thus, diverse experiments were performed by sonication of the solutions at various times in the range of 0.5–3.0 min. The obtained data (Fig. S2b†) depict that by increasing the sonication time to 1.0 min the efficiency of the method increases while the peak area decreases at higher times. Enhancing the method efficiency by increasing the sonication time is related to increasing the contacting time and area of the sample solution and biotin as the adsorbent. It is notable that decreasing the analytical data at times more than 10 min can be related to the back-extraction of the analyte from the sorbent surface into the sample solution. Subsequently, 1.0 min was utilized as the optimum extraction time.

### 3.4. Selection of elution solvent type and volume

**Fig. 3:**
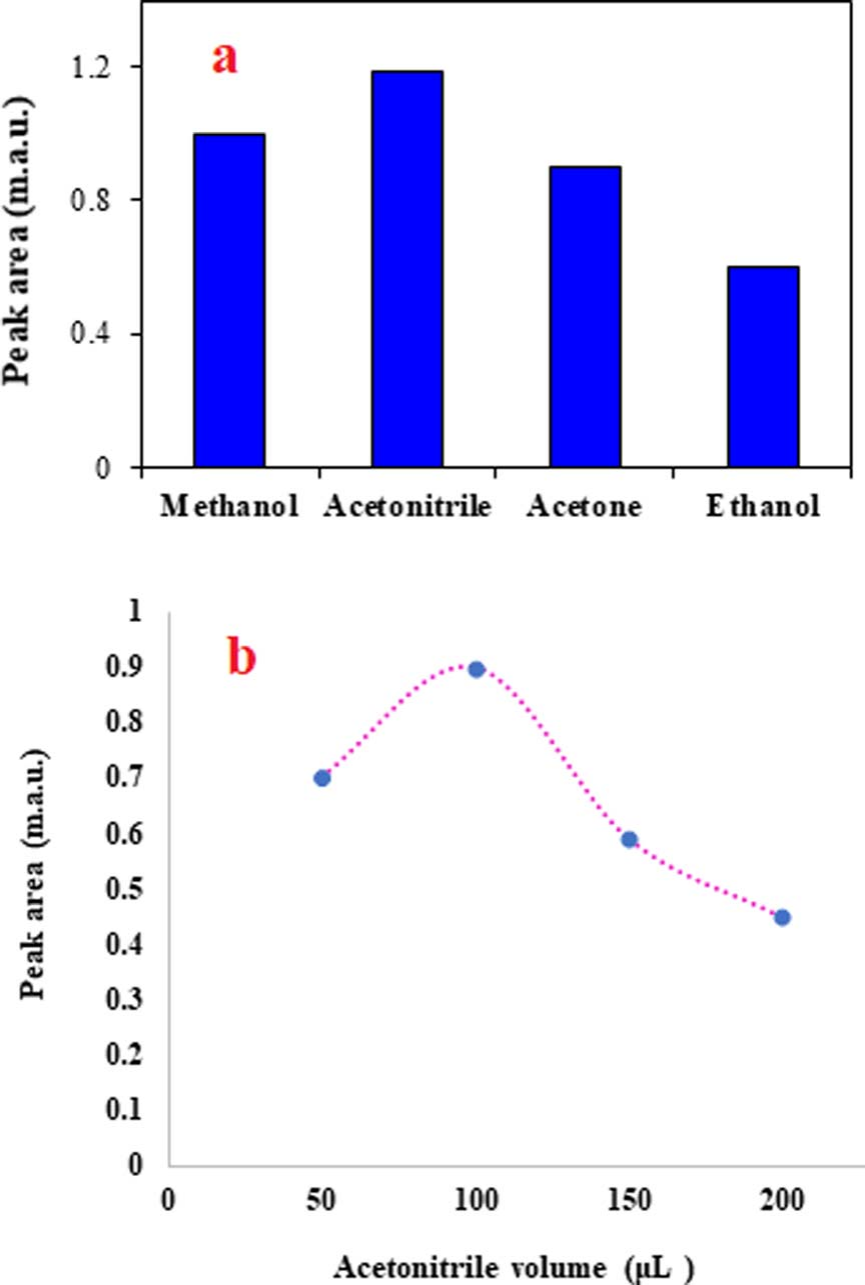
The effect of elution solvent type and volume on extraction efficiency. (a) Extraction conditions are the same as those discussed in the caption to Fig. 2b, except 1.0 min was selected as the optimum time of agitation. (b) Extraction conditions are the same as those discussed in the caption to Fig. 3a, except ACN was chosen as the eluent.

The solvent used to elute the adsorbed analyte from the adsorbent surface should have high solubility for the analyte. Also, among the candidate solvents to use as the elution solvent, in the present study, its solubility in the BGE is another basic factor for the selection of the elution solvent. Considering this factor, water-miscible solvents including methanol, ethanol, ACN, and acetone were tested. Although all tested solvents were able to desorb the analyte from the adsorbent surface, the results (Fig. 3a) showed that ACN is superior to the other solvents. The priority of ACN in the desorption of the analytes from the sorbent surface can be attributed to the higher solubility of the favipiravir in this solvent. Therefore, ACN was selected as the extraction solvent.

In addition to the type of the elution solvent, its volume affects the efficiency of the method. Higher volumes of the elution solvent may elute the analyte with better penetration into the solid adsorbent. Consequently, the volume of ACN was investigated by performing various tests in the range of 50–200 μL. It is noted that in all experiments, 100 μL of the eluent was taken and used in the analysis system. When the eluent volume was 50 μL, pure ACN was added to obtain a solution with a volume of 100 μL. The results shown in Fig. 3b confirm that the highest peak area was obtained for the analyte at 100 μL of ACN. It is obvious that dilution of the analyte at higher volumes of ACN leads to a decrease in the analytical signal. Therefore, ACN was selected for the next tests.

### 3.5. Salt effect

The addition of salt to the solution may enhance the efficiency of the DSPE method by decreasing the analyte's solubility in the solution. However, it can have an adverse effect on the extraction efficiency of the method by occupying the adsorption sites of the adsorbent or altering the diffusion coefficient of the analyte. To study the effect of salt on the method's efficiency, different concentrations of sodium chloride (0–5.0%, w/v) were added to the solution, and the method was performed on the solutions. The findings depicted in Fig. S3† indicate that as the sodium chloride concentration is gradually raised to 2.5% (w/v), the peak area of the analyte initially increases and subsequently decreases. Increasing the peak area of the analyte by dissolving NaCl up to 2.5% w/v is attributed to decreasing the analyte solubility in the solution and increasing the analyte's tendency to adsorb on the sorbent surface. Occupying the adsorption sites of the sorbent and increasing the solution migration rate are the major factors in decreasing the analytical data at more concentrations of NaCl (>2.5% w/v) As a result, all extractions were conducted using solutions containing 2.5% (w/v) of sodium chloride.

### 3.6. Optimization of desorption time

The effect of desorption time on the introduced method was evaluated by sonication of the mixture of the adsorbent and eluent at different times (in the range of 1–4 min). It was observed that with increasing sonication time up to 3 min, the method's efficiency increases and then reaches a constant level (Fig. S4†). It is clear that the penetration of the eluent into the sorbent effectively occurred at a high desorption time. Therefore, 3 min was selected as the optimum desorption time.

### 3.7. Method validation

To confirm the method's validity, several figures of merit were calculated according to ICH guidelines. Firstly, a calibration curve was plotted by preparing different solutions containing various concentrations of favipiravir and the peak area was plotted *versus* the concentration. The results showed that the curve has an acceptable coefficient of determination (*r*^2^ = 0.9968). The limit of detection and lower limit of quantification for the analyte by this method were calculated considering the signal (S) to noise (N) ratios of 3 and 10 and they were 75 and 250 ng mL^−1^, respectively. To examine the method's precision, a series of solutions were prepared and analyzed on the same day and various days. The relative standard deviations (RSDs) of the results were ≤8.2%. The extraction recovery of the method was obtained by dividing the amount of the extracted analyte by its initial concentration, which was 76%. The data are summarized in Table 1.

**Table 1:** Figures of merit of the developed method

Analyte	LOD^a^		LOQ^b^		LR^c^	*r* ^2^ d	RSD%^e^ at the concentration of				ER^f^
							250 ng mL^−1^		500 ng mL^−1^		
							Intra–day	Inter–day	Intra–day	Inter–day	
	In deionized water	In plasma and urine	In deionized water	In plasma and urine							
Favipiravir	60	300	200	1000	250–3000	0.9968	6.2	7.1	6.6	8.2	76

a Limit of detection (S/N = 3) (ng mL^−1^).

b Limit of quantification (S/N = 10) (ng mL^–1^).

c Linear range in deionized water (ng mL^–1^).

d Coefficient of determination.

e Relative standard deviation for intra–day (*n* = 5) and inter–day (*n* = 4) precisions in deionized water.

f Extraction recovery.

### 3.8. Selectivity, stability, matrix effect studies

In accordance with the ICH guidelines, ensuring the selectivity of an analytical method is paramount to its validation process. In our study, we rigorously evaluated the selectivity of our method by introducing additional antiviral drugs into both plasma and urine samples. Subsequently, we analyzed these samples using our method to determine if there were any interfering peaks that could potentially compromise the accuracy of the analyte's quantification. The results of this selectivity assessment demonstrated that there were no discernible interfering peaks that co-eluted with the analyte peak. This outcome underscores the high level of selectivity exhibited by our analytical method, confirming its ability to accurately detect and quantify the target analyte in the presence of other antiviral drugs. The stability of the analyte in the plasma sample was assessed by performing the method on spiked plasma and urine samples. These samples were stored at room temperature and in a freezer for 24 h. Additionally, we evaluated the analyte's stability in spiked samples subjected to multiple freezing and thawing cycles (*n* = 3). In all instances, the RSD values for the analyte, when compared to those of the freshly spiked samples at the same concentration, remained below 13%. This indicates that our analytical method demonstrates excellent stability under these conditions, ensuring reliable measurement of the analyte in both stored and freeze-thawed samples. In this approach, the utility of the method was accessed by the added-found method. For this purpose, blank urine and plasma samples were obtained from healthy volunteers, and they were spiked with the analyte at various concentrations . The slopes of calibration curves obtained in the samples and deionized water were statistically studied with respect to accuracy (*t*-test). The data illustrate that there is no significant difference between both cases. It verifies that the matrices of the samples have no significant effect on the analyte determination in the studied samples.

### 3.9. Comparison of the method with other approaches

The presented extraction procedure used for the determination of favipiravir in deionized water and biological samples were compared with other procedures considering the values related to LLOQ, LR, extraction time, extractant type and amount, and RSD%. Between the methods, the introduced method uses low or comparable amounts of extractant in comparison with other methods. The LR of this method is narrower than other methods. Extraction of the analytes can be performed in a shorter time by the developed method. The RSDs% of the method are comparable with those from other methods. All data are summarized in Table 2 in detail.

**Table 2:** Some analytical figures of merit of the proposed method compared to other methods

Sample	Extractant	LOQ a (ng mL^−1^)	LR^b^ (ng mL^−1^)	RSD^c^ (%)	Instrument	Extraction time (min)	Ref.
Plasma	900 μL of methanol and water	500	500–50000	<5.0	LC-MS/MS^d^	∼10	11
Plasma	30 mg of menthol	100	100–10000	<8.1	HPLC-UV e	∼20	14
Plasma	5 mL dichloromethane	300	300–60000	<3.0	HPLC-UV	∼15	4
Deionized water	30 mg biotin	200	250–3000	≤7.1	CE-DAD^f^	∼15	This work
Urine or plasma		1000					

a Limit of quantification.

b Linear range.

c Relative standard deviation.

d Liquid chromatography-tandem mass spectrometry.

e High performance liquid chromatography-ultraviolet detector.

f Capillary electrophoresis-diode array detector.

## 4. Conclusions

In the face of persistent global threats from infectious diseases, including the formidable challenge of emerging viral pathogens such as the novel coronavirus, the development of effective antiviral drugs remains a crucial endeavor. One such candidate in the fight against COVID-19 is favipiravir, a potent antiviral agent initially designed for influenza and subsequently explored for other viral pathogens, including Ebola and SARS-CoV-2. This study aimed to establish a robust and environmentally friendly method for the extraction of favipiravir from biological samples, such as urine and plasma, in preparation for its analysis *via* CE. The DSPE approach leverages an innovative strategy wherein the adsorbent, biotin, is generated *in situ* within the sample solution through an acid–base reaction, effectively capturing the analyte of interest.

Several critical optimization steps were undertaken to ensure the method's reliability and efficiency. The selection of the adsorbent, biotin, demonstrated its superior performance over folic acid. Additionally, optimizing the amount of biotin, the concentration and volume of HCl solution for pH adjustment, and the elution solvent type and volume are pivotal in achieving the method's high efficiency. The inclusion of sodium chloride at a concentration of 2.5% (w/v) proved beneficial for enhancing the extraction process.

Furthermore, the method's validation confirmed its precision, sensitivity, and selectivity. The recovery experiments using real urine and plasma samples substantiated the method's accuracy and negligible matrix effects. The developed DSPE method offers a reliable and environmentally friendly approach for pharmacokinetic studies and therapeutic monitoring.

## Abbreviations

DSPE: Dispersive solid phase extraction
CE: Capillary electrophoresis
ER: Extraction recovery
LOD: Limit of detection
LOQ: Limit of quantification
RSD: Relative standard deviation
LR: Linear range

## Conflicts of interest

The authors have declared no conflict of interest.

## Supplementary Material

RA-014-D3RA07356D-s001
