# Supplementary material for: Acid–base reaction-based dispersive solid phase extraction of favipiravir using biotin from biological samples prior to capillary electrophoresis analysis
Source: RSC Adv. 2024 Jun 19;14(27):19612–8. doi: 10.1039/d3ra07356d (PMC11188667; doi:10.1039/d3ra07356d)
Supplement: RA-014-D3RA07356D-s001 [file RA-014-D3RA07356D-s001.pdf]

Electronic Supplementary Material (ESI) for RSC Advances.
This journal is © The Royal Society of Chemistry 2024

Supplementary data

Acid-base reaction-based dispersive solid phase extraction of favipiravir using biotin from biological samples prior to capillary electrophoresis analysis

Elnaz Safari[a], Behrouz Seyfinejad[b,c], Mir Ali Farajzadeh[d,e], Mohammad Reza Afshar Mogaddam[c,*] Mahboob Nemati[a,c*]

[a] *Pharmaceutical and Food Control Department, Faculty of Pharmacy, Tabriz University of Medical Sciences, Tabriz, Iran*

[b] *Pharmaceutical Analysis Research Center, Tabriz University of Medical Sciences, Tabriz, Iran*

[c] *Food and Drug Safety Research Center, Tabriz University of Medical Sciences, Tabriz, Iran*

[d] *Department of Analytical Chemistry, Faculty of Chemistry, University of Tabriz, Tabriz, Iran*

[e] *Engineering Faculty, Near East University, 99138 Nicosia, North Cyprus, Mersin 10, Turkey*

*Corresponding author: Prof. M. Nemati

Email address: nematim@tbzmed.ac.ir; mahnema@gmail.com

*Corresponding author: Dr. M. R. Afshar Mogaddam

Email address: mr.afsharmogaddam@yahoo.com; Afsharmogaddam@tbzmed.ac.ir

Tel.: +98 4133372250

Fax: 98 4133344798

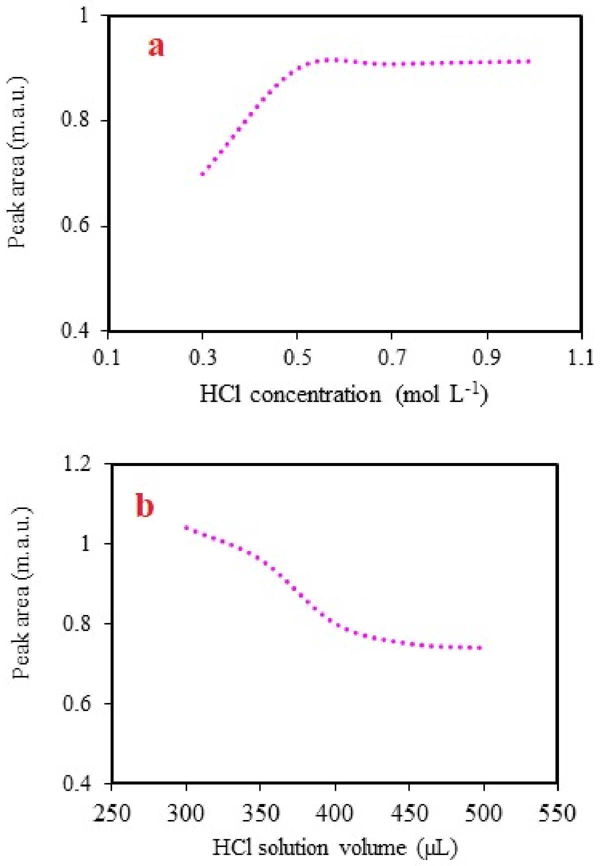

Fig. S1. The effect of HCl solution concentration (a) and volume (b) on extraction efficiency.

(a) Extraction conditions: sample, 5 mL deionized water spiked with favipiravir at 250 ng mL^{-1}; adsorbent (amount), biotin (25 mg); acid solution volume, 300 μL; agitation type (time), vortex (1 min); elution solvent (volume), ACN (200 μL); desorption time, 2 min; and centrifugation rate (time), 4000 rpm, 5 min. (b) Extraction conditions: are the same as those used in Fig. S1a, except the HCl solution concentration was 0.5 mol L^{-1}.

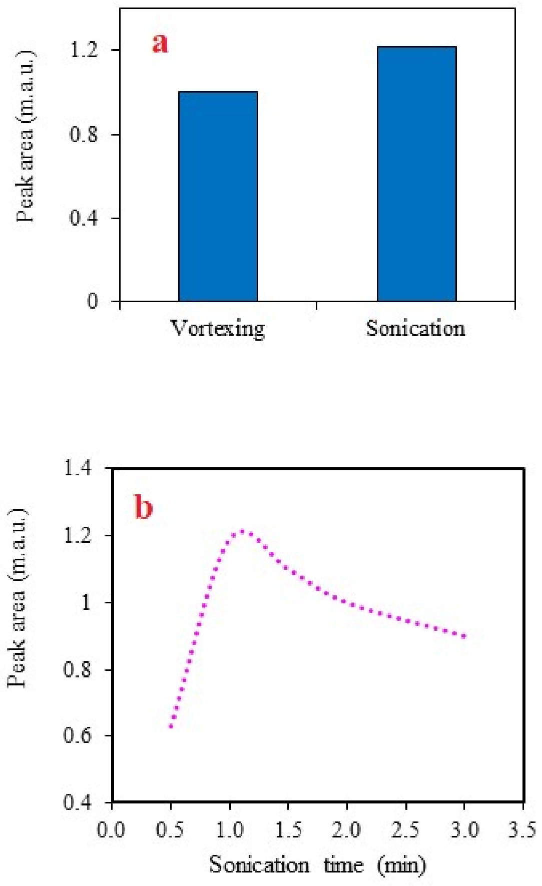

Fig. S2. The effect of agitation type (a) and time (b) on extraction efficiency.

(a) Extraction conditions: are the same as those used in Fig. S1b, except 300 μL of HCl solution (0.5 mol L^{-1}) was used.

(b) Extraction conditions: are the same as those used in Fig. S2a, except sonication was selected as the agititation type.

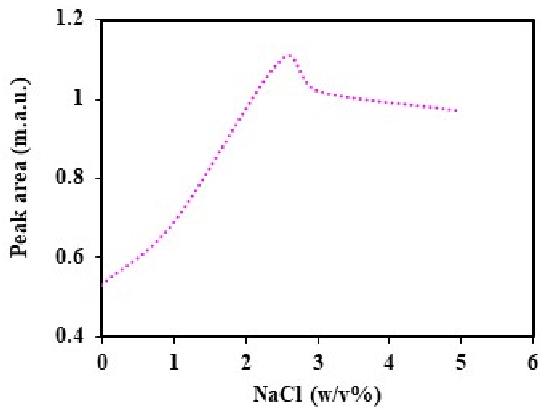

Fig. S3. The effect of salt addition on extraction efficiency.

Extraction conditions: are the same as those used in Fig. 4b, except 100 µL of ACN and 1 min were chosen as the eluent and adsorption time, repetively.

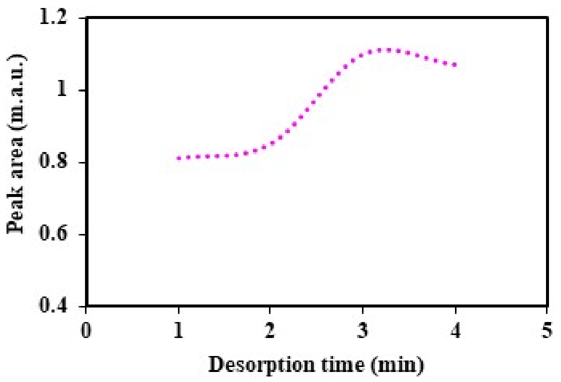

Fig. S4. The effect of desorption time on extraction efficiency.

Extraction conditions: are the same as those used in Fig. S3, except 2.5% (w/v) NaCl was dissolved in the solution.
